# Clinical characteristics of non-idiopathic pulmonary fibrosis, progressive fibrosing interstitial lung diseases: A single-center retrospective study

**DOI:** 10.1097/MD.0000000000025322

**Published:** 2021-04-02

**Authors:** Masamichi Komatsu, Hiroshi Yamamoto, Yoshiaki Kitaguchi, Satoshi Kawakami, Mina Matsushita, Takeshi Uehara, Takumi Kinjo, Yosuke Wada, Takashi Ichiyama, Kazuhisa Urushihata, Atsuhito Ushiki, Masanori Yasuo, Masayuki Hanaoka

**Affiliations:** a First Department of Internal Medicine; b Department of Radiology; c Department of Laboratory Medicine, Shinshu University School of Medicine, Matsumoto, Japan.

**Keywords:** idiopathic pulmonary fibrosis, interstitial lung disease, progressive fibrosing interstitial lung disease

## Abstract

Progressive fibrosing interstitial lung disease (PF-ILD) is a progressive phenotype of fibrosing ILDs with varying definitions and elusive clinical characteristics. We aimed to clarify the clinical features and prognosis of PF-ILD cases based on the deterioration of pulmonary function.

Altogether, 91 consecutive ILD patients who underwent at least 2 pulmonary function tests (PFTs) with an interval of at least 24 months, as the screening period, between January 2009 and December 2015 were retrospectively reviewed. The deterioration of forced vital capacity (FVC) and diffusing capacity of the lung for carbon monoxide (DLco) was calculated based on PFT data and screening period. The definition of PF-ILD was

1. relative decline of 10% or more in FVC per 24 months or
2. relative decline in FVC of 5% or more with decline in DLco of 15% or more per 24 months.

Medical records of 34 patients with idiopathic pulmonary fibrosis (IPF), 11 patients with non-IPF, PF-ILD, and 46 patients with non-IPF, non-PF-ILD were retrospectively analyzed. Patient characteristics, pharmacologic or non-pharmacologic treatment status, and prognosis were compared between the IPF and non-IPF groups and between the non-IPF, PF-ILD and non-IPF, non-PF-ILD groups.

Eleven patients (19.3%) showed a progressive phenotype in the non-IPF group. The pulmonary function data at the first PFT were worse in non-IPF, PF-ILD patients than in non-IPF, non-PF-ILD patients. There were no differences in the proportion of patients who were observed without pharmacologic treatment or of those receiving pharmacologic treatment between the non-IPF, PF-ILD and non-IPF, non-PF-ILD groups. Low %FVC at the first PFT and the usual interstitial pneumonia-like fibrotic pattern on high-resolution computed tomography were risk factors for PF-ILD in the non-IPF group. The mortality in the non-IPF, PF-ILD group was significantly worse than that of the non-IPF, non-PF-ILD group and was as poor as that of the IPF group. Multivariate logistic analysis showed that aging and low %DLco at the first PFT were risk factors for mortality within the non-IPF group.

The prognosis of non-IPF, PF-ILD patients was as poor as that of IPF patients. Non-IPF, PF-ILD patients require more intensive treatment before disease progression.

## 1 Introduction

Idiopathic pulmonary fibrosis (IPF), the most common form of idiopathic interstitial pneumonia (IIPs), is a chronic and progressive fibrosing interstitial lung disease (ILD) of unknown etiology.^[1–3]^ The median survival period of IPF is 2 to 3.5 years, and patients have a poor prognosis.^[4–6]^ However, other forms of ILD besides IPF,^[7]^ including other IIPs,^[8,9]^ chronic hypersensitivity pneumonitis,^[10]^ and connective tissue disease (CTD)-related ILD (CTD-ILD),^[11]^ can also develop a progressive phenotype. Recently, these fibrosing ILDs were grouped under the term “progressive fibrosing ILDs” (PF-ILDs).^[12]^

The definitions of disease progression of ILD vary across studies.^[13]^ In most clinical trials or observational studies, the rate of decline in forced vital capacity (FVC) was used to assess disease progression in ILD patients.^[14–16]^ Furthermore, gas exchange parameters, including the diffusing capacity of the lung for carbon monoxide (DLco), worsening of symptoms, exercise capacity, deterioration in health-related quality of life, the extent of lung fibrosis on high-resolution computed tomography (HRCT), or the initiation of supplemental oxygen therapy, were used to assess disease progression.^[12]^

In INBUILD trials,^[16]^ non-IPF, PF-ILD was defined as a relative decline in FVC, worsening of symptoms, or increased extent of fibrotic changes on chest HRCT within 24 months. Nintedanib demonstrated slowing disease progression in non-IPF, PF-ILD in INBUILD trials,^[16]^ and now, nintedanib is one of the essential treatment options in ILDs with a progressive phenotype. However, the clinical features of non-IPF, PF-ILD in a real-world setting remain unknown.

In this study, we retrospectively analyzed the clinical features of non-IPF, PF-ILD patients based on the deterioration of their pulmonary function in the past 24 months in a real-world setting.

## 2 Methods

### 2.1 Study subjects

This study was approved by the Ethics Committee of the Shinshu University School of Medicine (Approval Number 4591) and was performed in accordance with the Declaration of Helsinki and its subsequent amendments. The need for obtaining patient written informed consent was waived, owing to the retrospective nature of this study.

**Figure 1:**
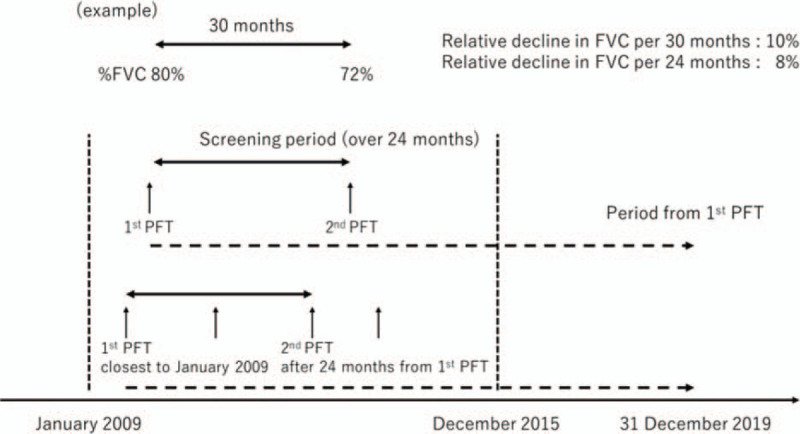
Definition of the first and second PFTs. FVC = forced vital capacity, PFT = pulmonary function test.

This retrospective, single-center study reviewed the medical records of ILD patients, including those with IIPs, CTD-ILDs, and other related ILDs, such as drug-induced lung disease, who underwent at least 2 pulmonary function tests (PFTs) with an interval of at least 24 months, as the screening period, at the Shinshu University Hospital between January 2009 and December 2015. For patients who underwent multiple PFTs during the same period, the PFT performed closest to January 2009 was defined as the first PFT, and that performed at 24 months after the first PFT was defined as the second (Fig. 1). In this study, patients with ILD who underwent surgical lung resection during the screening period were excluded.

The clinical, radiological, and pathological findings and disease behaviors were reviewed, and ILD diagnoses were made in a multidisciplinary discussion. IPF was diagnosed according to the 2018 Clinical Practice Guideline.^[2]^ IIPs other than IPF were diagnosed based on the 2013 Official Statement of the American Thoracic Society/European Respiratory Society.^[3]^ The CTD-ILD patients fulfilled each of the standard criteria.^[17–22]^ Patients showing anti-aminoacyl-tRNA synthetase autoantibodies without polymyositis/dermatomyositis were diagnosed as having antisynthetase syndrome associated ILDs. Patients with anti-neutrophil cytoplasmic antibodies (ANCA) were diagnosed as having ANCA-associated ILDs.^[23,24]^ Patients with sarcoidosis and drug-induced pneumonitis were diagnosed according to the respective criteria.^[25,26]^ In this study, PF-ILD was defined only by the deterioration of pulmonary function, in consideration of data loss such as subjective symptoms or deterioration of fibrosis on HRCT. In particular, PF-ILD was defined as the

1. relative decline of 10% or more in FVC per 24 months and
2. the relative decline in FVC of 5% or more with decline in DLco of 15% or more per 24 months, with reference to recently suggested definitions.^[27]^

**Figure 2:**
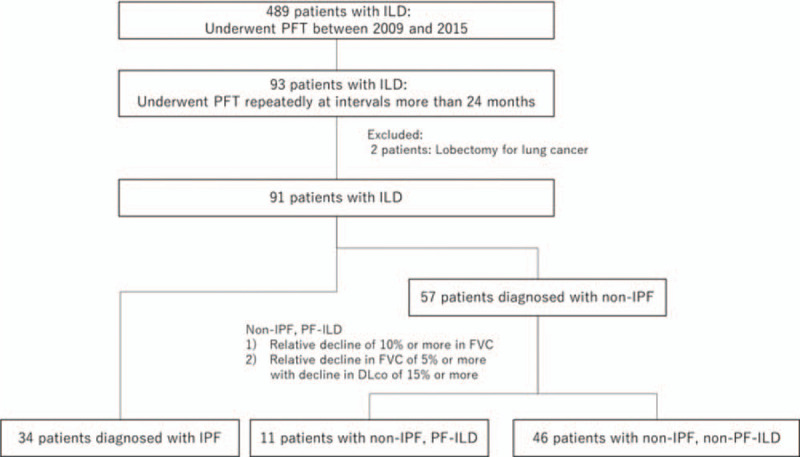
Flow diagram of patient selection. FVC = forced vital capacity, ILD = interstitial lung disease, IPF = idiopathic pulmonary fibrosis, PF = progressive fibrosing, PFT = pulmonary function test.

A flow diagram showing patient selection is presented in Figure 2. We screened 489 consecutive patients who underwent PFT between January 2009 and December 2015. In total, 93 patients underwent PFT repeatedly, at intervals greater than 24 months. Two patients were excluded because they underwent surgical lung resection during the screening period. Finally, 34 and 57 patients with IPF and non-IPF, respectively, were enrolled in this study.

### 2.2 Radiographic evaluation

Two radiologists (S.K. and M.M.) independently reviewed the chest HRCT findings during the first PFT and determined whether the patient showed a usual interstitial pneumonia (UIP)-like fibrotic pattern or a non-UIP pattern without referring to clinical information. In case of disagreement on HRCT findings between the 2 reviewers, they discussed and resolved the case by mutual consensus. The UIP-like fibrotic pattern was defined according to the radiological criteria in the INPULSIS trials.^[14]^ When the HRCT findings met criteria A, B, and C as described below, or criteria A and C or B and C, the HRCT pattern was defined as a UIP-like fibrotic pattern. These HRCT criteria were as follows: A) definite honeycomb lung destruction with basal and peripheral predominance; B) presence of reticular abnormality and traction bronchiectasis consistent with fibrosis, with basal and peripheral predominance; and C) absence of atypical features, specifically nodules and consolidation. Ground glass opacity, if present, was less extensive than the reticular opacity pattern.

### 2.3 PFTs and relative decline in FVC and DLco

In this study, patients underwent PFTs, including spirometry and measurements of the DLco, functional residual capacity, total lung capacity (TLC), and residual volume, using a pulmonary function testing system (Chestac-8900; Chest Co. Ltd, Tokyo, Japan), as previously reported.^[28,29]^ The composite physiologic index (CPI),^[30,31]^ representing a combination of pulmonary ventilation and diffusing capacity, was also calculated based on the pulmonary function data.

Relative decline in FVC and DLco per 24 months was calculated based on PFT data and screening period. For example, when the percent predicted FVC (%FVC) at the first PFT was 80% and that at the second PFT 30 months later was 72%, the relative decline in FVC per 30 months was calculated as 10%, and the relative decline in FVC per 24 months was calculated as 8% (Fig. 1).

### 2.4 Statistical analysis

Continuous data are presented as median and range, and categorical data are presented as the number in each group. While the Mann–Whitney *U* test or unpaired *t*-test was used to compare continuous variables between the 2 groups, the χ^2^ test or the Fisher exact test was used to compare categorical variables. A multivariate logistic regression analysis was performed to verify the risk for PF-ILD in the non-IPF group. Univariate Cox proportional hazards regression analysis followed by multivariate analysis was used to identify the risk factors associated with mortality in the non-IPF group. The overall survival, median, and 95% confidence intervals were determined using the Kaplan–Meier method, whereas intergroup differences were compared using the log-rank test. The cut-off date for the observation period was December 31, 2019. Statistical analyses were performed using StatFlex Version 7.0 (Artech, Osaka, Japan). Statistical significance was established at *P* values of <.05.

## 3 Results

### 3.1 Clinical characteristics of the IPF and non-IPF groups

The clinical characteristics of the IPF and non-IPF patients are described in Table 1. The IPF patients were significantly older than the non-IPF patients at the first PFT (*P* = .04). The respective pulmonary function data and CPI at the first PFT in the IPF and non-IPF groups did not differ significantly, except for percent predicted TLC (%TLC). The %TLC and %DLco at the second PFT were poorer in the IPF group than in the non-IPF group (*P* < .01, and *P* < .01, respectively). CPI at the second PFT was also poorer in the IPF group than in the non-IPF group (*P* < .01). Relative decline in FVC ≥10% per 24 months was observed in 6 (17.6%) patients in the IPF group and 9 (15.8%) patients in the non-IPF group. Relative decline in DLco ≥15% per 24 months was observed in 17 (50.0%) patients in the IPF group and 11 (19.3%) patients in the non-IPF group.

**Table 1:** Clinical characteristics of the IPF and non-IPF groups.

	IPF (n = 34)	Non-IPF (n = 57)	*P*
Age at the first PFT, (yr)	69.5 (45.0–80.0)	65.0 (32.0-86.0)	.04
Sex			.02
Male, n (%)	28 (82.4)	33 (57.9)	
Female, n (%)	6 (17.6)	24 (42.1)	
Smoking status			< .01
Never, n (%)	6 (17.6)	29 (50.9)	
Ever/Current, n (%)	28 (82.4)	28 (49.1)	
HRCT pattern			< .01
UIP-like fibrotic pattern, n (%)	34 (100)	11 (19.3)	
Non-UIP pattern, n (%)	0 (0.0)	46 (80.7)	
Laboratory data			
Total protein, (g/dl)	7.6 (6.5–10.1)	7.4 (6.1–10.1)	.79
Albumin, (g/dl)	4.2 (3.6–4.7)	4.1 (2.9–4.8)	.01
LDH, (U/L)	213 (150–457)	207 (96–513)	.76
C-reactive protein, (mg/dl)	0.13 (0.02–5.13)	0.15 (0.00–7.85)	.41
KL-6, (U/ml)	717 (167–2283)	797 (165–8020)	.13
Pulmonary function (at the first test)			
FVC, (L)	2.84 (1.37–4.03)	2.64 (1.31–5.05)	.67
%FVC, (%)	87.2 (43.0–119.2)	84.5 (49.0–130.6)	.92
TLC, (L)	4.51 (1.91–6.55)	4.27 (2.35–8.42)	.47
%TLC, (%)	90.7 (53.7–125.2)	94.6 (50.4–165.3)	.03
DLco, (ml/min/mm Hg)	13.8 (5.4–21.1)	13.5 (4.0–28.2)	.43
%DLco, (%)	57.6 (27.7–83.0)	62.5 (16.3–107.3)	.30
CPI	36.21 (11.27–63.51)	32.96 (-11.45–66.89)	.18
Pulmonary function (at the second test)			
FVC, (L)	2.70 (1.29–4.26)	2.64 (0.82–5.10)	.69
%FVC, (%)	84.8 (38.1–127.9)	86.2 (30.1–131.8)	.32
TLC, (L)	3.97 (2.18–6.62)	4.15 (2.14–7.52)	.14
%TLC, (%)	80.2 (46.0–127.1)	97.1 (48.5–135.0)	< .01
DLco, (ml/min/mm Hg)	11.5 (3.7–20.3)	13.8 (4.3–32.4)	.01
%DLco, (%)	48.5 (16.3–86.8)	60.8 (18.8–113.9)	< .01
CPI	44.03 (2.22–84.75)	33.17 (-13.66–83.04)	< .01
Screening period, (months)	28.5 (24.0–54.0)	30.0 (24.0–52.0)	.30
Deterioration in pulmonary function			
Relative decline in FVC ≧ 10%, n (%)	6 (17.6%)	9 (15.8%)	.95
Relative decline in FVC 10>, ≧ 5%, n (%)	5 (14.7%)	8 (14.0%)	.82
Relative decline in DLco ≧ 15%, n (%)	17 (50.0%)	11 (19.3%)	< .01
Observation period, (yr)	6.44 (2.20–10.51)	7.67 (2.02-10.76)	.09
Acute exacerbation, n (%)	7 (20.6%)	4 (7.0%)	.09

Data are presented as median (range) or number (%). CPI = composite physiologic index, DLco = diffusing capacity of the lung for carbon monoxide, FVC = forced vital capacity, HRCT = high-resolution computed tomography, IPF = idiopathic pulmonary fibrosis, KL-6 = Krebs von den Lungen-6, LDH = lactate dehydrogenase, PFT = pulmonary function test, TLC = total lung capacity, UIP = usual interstitial pneumonia.

### 3.2 Clinical characteristics of the non-IPF, PF-ILD and non-IPF, non-PF-ILD groups

Patients with non-IPF were further divided into the non-IPF, PF-ILD and non-IPF, non-PF-ILD groups. Eleven of 57 (19.3%) patients with non-IPF showed a progressive phenotype (9 patients showed the relative decline of 10% or more and 2 patients showed the relative decline in FVC of 5% or more with decline in DLco of 15% or more per 24 months). Clinical characteristics of the non-IPF, PF-ILD and non-IPF, non-PF-ILD patients are described in Table 2. The UIP-like fibrotic pattern on HRCT was more dominant in the non-IPF, PF-ILD group than in the non-IPF, non-PF-ILD group (*P* = .03). In the non-IPF group, 2 of the 7 patients (28.6%) with idiopathic non-specific interstitial pneumonia (iNSIP), 2 of the 16 patients (12.5%) with unclassifiable IIPs, and 3 of the 22 patients (13.3%) with CTD-ILD were diagnosed with non-IPF, PF-ILD. The %FVC and %TLC at the first PFT in the non-IPF, PF-ILD group were poorer than those of the non-IPF, non-PF-ILD group. Additionally, pulmonary function data and CPI at the second PFT in the non-IPF, PF-ILD group were also poorer than those of the non-IPF, non-PF-ILD group.

**Table 2:** Clinical characteristics of the non-IPF, PF-ILD and non-IPF, non-PF-ILD groups.

	Non-IPF, PF-ILD (n = 11)	Non-IPF, non-PF-ILD (n = 46)	*P*
Age at the first PFT, (yr)	67.0 (37.0–83.0)	65.0 (32.0–86.0)	.46
Sex			.50
Male, n (%)	5 (45.5%)	28 (60.9%)	
Female, n (%)	6 (54.5%)	18 (39.1%)	
Smoking status			.11
Never, n (%)	3 (27.3%)	25 (54.8%)	
Ever/Current, n (%)	8 (72.7%)	21 (45.7%)	
HRCT pattern			.03
UIP-like fibrotic, n (%)	5 (54.5%)	6 (13.0%)	
Non-UIP pattern, n (%)	6 (45.5%)	40 (87.0%)	
Diagnosis			
Idiopathic NSIP, n (%)	2 (18.2%)	5 (10.9%)	
Unclassifiable IIPs, n (%)	2 (18.2%)	14 (30.4%)	
PPFE, n (%)	2 (18.2%)	0 (0.0%)	
CTD-ILD, n (%)	3 (27.3%)	19 (41.3%)	
SSc associated ILD, n	2	4	
Sjogren's syndrome-associated ILD, n	0	6	
RA-associated ILD, n	0	3	
PM/DM-associated ILD, n	0	3	
Sjogren's syndrome and ASS-associated ILD, n	1	2	
SLE-associated ILD, n	0	1	
ANCA-associated ILD, n (%)	1 (9.1%)	1 (2.2%)	
Sarcoidosis, n (%)	0	4 (11.1%)	
Other ILD, n (%)	1 (9.1%)	3 (6.5%)	
Laboratory data			
Total protein, (g/dl)	7.3 (6.4–8.1)	7.2 (5.5–10.2)	.78
Albumin, (g/dl)	3.8 (3.3–4.4)	3.9 (2.1–5.3)	.26
LDH, (U/L)	234 (199–346)	206 (95–444)	< .01
C-reactive protein, (mg/dl)	0.20 (0.03–2.18)	0.10 (0.00–11.34)	.33
KL-6, (U/ml)	766 (205–4689)	499 (166–4409)	.22
Pulmonary function (at the first test)			
FVC, (L)	2.08 (1.31–3.69)	2.71 (1.59–5.05)	.02
%FVC, (%)	77.9 (49.0–101.9)	88.2 (57.2–130.6)	.02
TLC, (L)	3.18 (2.35–6.13)	4.72 (2.45–8.42)	.02
%TLC, (%)	81.1 (50.4–112.7)	100.4 (61.3–165.3)	.02
DLco, (ml/min/mm Hg)	12.1 (4.0–19.9)	14.4 (4.1–28.2)	.21
%DLco, (%)	56.7 (17.7–82.3)	64.0 (16.3–107.3)	.36
CPI	37.5 (8.0–66.9)	30.0 (−11.4–61.3)	.15
Pulmonary function (at the second test)			
FVC, (L)	1.67 (0.82–3.05)	2.74 (1.40–5.10)	< .01
%FVC, (%)	60.7 (30.1–95.1)	91.4 (54.0–131.8)	< .01
TLC, (L)	2.78 (2.14–6.23)	4.56 (2.14–7.52)	< .01
%TLC, (%)	71.7 (48.5–115.4)	99.3 (56.6–135.0)	< .01
DLco, (ml/min/mm Hg)	9.7 (4.3–19.8)	14.7 (5.0–32.4)	.04
%DLco, (%)	45.4 (18.8–83.2)	64.8 (22.0–113.9)	.06
CPI	53.6 (16.7–83.0)	29.2 (−13.7–64.1)	< .01
Screening period, (mo)	29.0 (24.0–51.0)	30.0 (24.0–52.0)	.95
Observation period, (yr)	5.45 (2.75–10.17)	8.09 (2.02–10.76)	.02
Acute exacerbation, n (%)	1 (9.1%)	3 (6.5%)	.72

Data are presented as median (range) or number (%). ANCA = anti-neutrophil cytoplasmic antibody, ASS = anti-synthetase syndrome, CPI = composite physiologic index, CTD = connective tissue disease, DLco = diffusing capacity of the lung for carbon monoxide, DM = dermatomyositis, FVC = forced vital capacity, HRCT = high-resolution computed tomography, IIP = idiopathic interstitial pneumonia, ILD = interstitial lung disease, IPF = idiopathic pulmonary fibrosis, KL-6 = Krebs von den Lungen-6, LDH = lactate dehydrogenase, NSIP = non-specific interstitial pneumonia, PF = progressive fibrosing, PFT = pulmonary function test, PM = polymyositis, PPFE = pleuroparenchymal fibroelastosis, RA = rheumatoid arthritis, SLE = systemic lupus erythematodes, SSc = systemic sclerosis, TLC = total lung capacity, UIP = usual interstitial pneumonia.

We examined the risk factors for PF-ILD in 57 non-IPF patients (Table 3). The logistic regression analysis showed that a UIP-like fibrotic pattern on HRCT and low %FVC at the first PFT were associated with a high risk of PF-ILD in the non-IPF group (*P* = .02 and *P* = .03, respectively). However, in the multivariate analysis, these were not significant risk factors for PF-ILD in the non-IPF group.

**Table 3:** Logistic regression analysis investigating the risk factors for PF-ILD in the non-IPF group.

	Univariate			Multivariate		
	Odds ratio	95% CIs	*P*	Odds ratio	95% CIs	*P*
Age at the first PFT	1.022	0.965–1.082	.45			
Male	0.536	0.142–2.018	.36			
UIP-like fibrotic pattern on HRCT	5.556	1.284–24.030	.02	3.659	0.771–17.368	.10
%FVC at the first PFT	0.955	0.916–0.996	.03	0.964	0.923–1.008	.11
%DLco at the first PFT	0.986	0.956–1.016	.35			

CI = confidence interval, DLco = diffusing capacity of the lung for carbon monoxide, FVC = forced vital capacity, HRCT = high-resolution computed tomography, ILD = interstitial lung disease, IPF = idiopathic pulmonary fibrosis, PF-ILD = progressive fibrosing interstitial lung disease, PFT = pulmonary function test, UIP = usual interstitial pneumonia.

### 3.3 Treatment status during the overall study period

Treatment status throughout the study period for each disease of the IPF and non-IPF groups are shown in Table 4, and each disease of the non-IPF, PF-ILD and non-IPF, non-PF-ILD groups are shown in Table 5. Antifibrotic agents were administered more frequently in the IPF group, whereas immunosuppressant agents were administered more frequently in the non-IPF group. There were no differences in the proportion of patients undergoing observation without pharmacologic treatment and the proportion of patients receiving pharmacologic treatment with corticosteroids or immunosuppressant agents between the non-IPF, PF-ILD and non-IPF, non-PF-ILD groups. Six of 11 patients with non-IPF, PF-ILD underwent observation without pharmacologic treatment despite the deterioration. Supplemental oxygen was more likely to be administered in the non-IPF, PF-ILD group than in the non-IPF, non-PF-ILD group.

**Table 4:** Treatment status in the IPF and non-IPF groups.

	Univariate			Multivariate		
	Odds ratio	95% CIs	*P*	Odds ratio	95% CIs	*P*
Age at the first PFT	1.022	0.965–1.082	.45			
Male	0.536	0.142–2.018	.36			
UIP-like fibrotic pattern on HRCT	5.556	1.284–24.030	.02	3.659	0.771–17.368	.10
%FVC at the first PFT	0.955	0.916–0.996	.03	0.964	0.923–1.008	.11
%DLco at the first PFT	0.986	0.956–1.016	.35			

Data are presented as number (%). IPF = idiopathic pulmonary fibrosis.

**Table 5:** Treatment status in the non-IPF, PF-ILD and non-IPF, non-PF-ILD groups.

	Non-IPF, PF-ILD (n = 11)	Non-IPF, non-PF-ILD (n = 46)	*P*
Observation without pharmacologic treatment, n (%)	6 (54.5%)	25 (54.3%)	.99
Pharmacologic treatment			
Corticosteroids, n (%)	4 (36.4%)	19 (41.3%)	.97
Immunosuppressant agents, n (%)	2 (18.2%)	10 (21.7%)	.88
Antifibrotic agents, n (%)	1 (9.1%)	0 (0.0%)	.43
Nonpharmacologic treatment			
Long-term oxygen therapy, n (%)	6 (54.5%)	13 (28.3%)	.15

Data are presented as number (%). IPF = idiopathic pulmonary fibrosis, PF-ILD = progressive fibrosing interstitial lung disease.

### 3.4 Survival analysis by disease group

**Figure 3:**
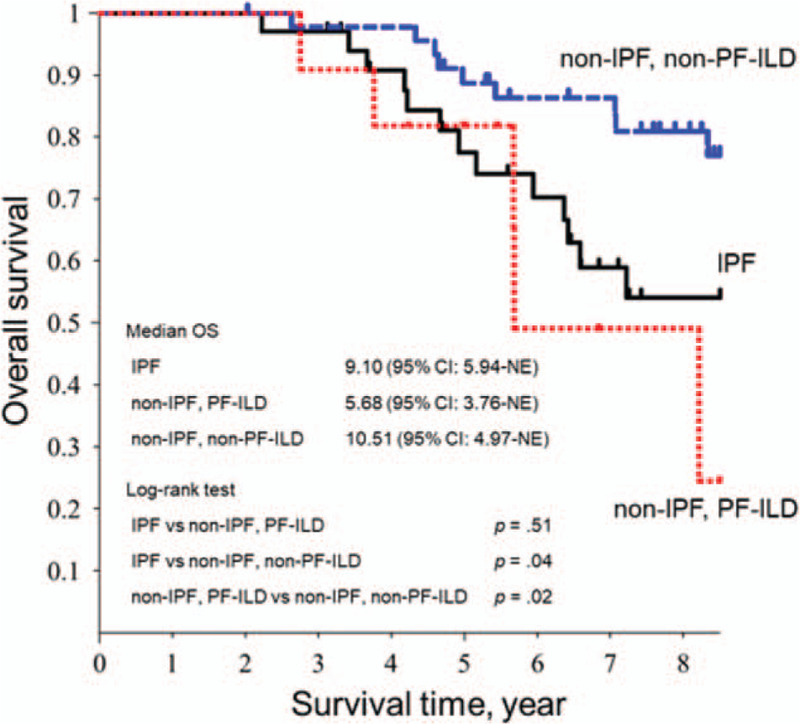
Survival curves of the IPF, non-IPF, PF-ILD, and non-IPF, non-PF-ILD groups. The Kaplan–Meier curve of overall survival in the IPF, non-IPF, PF-ILD, and non-IPF, non-PF-ILD groups (black: IPF group, red: non-IPF, PF-ILD group, blue: non-IPF, non-PF-ILD group). CI = confidence interval, ILD = interstitial lung disease, IPF = idiopathic pulmonary fibrosis, NE = not evaluable, OS = overall survival, PF = progressive fibrosing.

The Kaplan–Meier curves of overall survival in each disease group from the first PFT are shown in Figure 3. Survival analysis demonstrated that the IPF group had a significantly worse survival than the non-IPF, non-PF-ILD group (*P* = .02). Similarly, the non-IPF, PF-ILD group had a significantly worse survival than the non-IPF, non-PF-ILD group (*P* = .01). A similar trend was demonstrated in the survival analysis between the IPF and non-IPF, PF-ILD groups.

We identified the risk factors for mortality in 57 non-IPF patients by performing a Cox proportional hazards regression analysis (Table 6). The univariate analysis revealed that aging, low %DLco at the first PFT, and PF-ILD were significant risk factors for mortality (*P* < .01, *P* = .02, and *P* = .03, respectively). In a multivariate analysis, aging and low %DLco at the first PFT were independent risk factors for mortality (*P* = .03, and *P* = .02, respectively).

**Table 6:** Cox proportional hazards regression analysis investigating the risk factors for mortality in the non-IPF group.

	Univariate			Multivariate		
	Hazard ratio	95% CIs	*P*	Hazard ratio	95% CIs	*P*
Age at the first PFT	1.084	1.025–1.147	< .01	1.069	1.007–1.136	.03
Male	2.262	0.768–6.666	.14			
UIP-like fibrotic pattern on HRCT	2.470	0.843–7.243	.10			
%FVC at the first PFT	0.973	0.946–1.000	.05			
%DLco at the first PFT	0.970	0.947–0.994	.02	0.967	0.940–0.995	.02
PF-ILD	3.442	1.146–10.336	.03	2.353	0.713–7.763	.16

CI = confidence interval, DLco = diffusing capacity of the lung for carbon monoxide, FVC = forced vital capacity, HRCT = high-resolution computed tomography, IPF = idiopathic pulmonary fibrosis, PF-ILD = progressive fibrosing interstitial lung disease, PFT = pulmonary function test, UIP = usual interstitial pneumonia.

## 4 Discussion

This study showed 3 main findings regarding the clinical characteristics of patients with non-IPF, PF-ILD. First, the pulmonary function data at the first PFT were worse in the non-IPF, PF-ILD than in the non-IPF, non-PF-ILD group. Second, approximately half of patients with non-IPF, PF-ILD underwent observation without pharmacologic treatment, despite the deterioration of their pulmonary function. Last, the prognosis of the non-IPF, PF-ILD group was significantly worse than that of the non-IPF, non-PF-ILD group and was as poor as that of the IPF group.

The term “PF-ILD” describes chronic fibrosing ILDs and mostly includes IPF, iNSIP,^[32]^ unclassifiable ILD,^[9]^ CTD-ILDs, including rheumatoid arthritis-associated ILD,^[33]^ systemic sclerosis-associated ILD,^[34]^ chronic hypersensitivity pneumonitis,^[35]^ and exposure-related disease.^[12,13]^ The definition of PF-ILD varies across studies; therefore, there is no consensus on the definition of disease progression in patients with ILDs. Nevertheless, FVC decline has been used in some trials for IPF patients,^[14,36]^ and is a well-established predictor of mortality.^[37]^ A recent phase III study, the INBUILD trial,^[16]^ demonstrated slower disease progression in non-IPF, PF-ILD patients with nintedanib treatment. This trial recruited non-IPF patients showing diffuse fibrosing lung disease with >10% extent of fibrosis on HRCT scans who met the criteria for disease progression at 24 months before screening, based on a relative decline of 10% or more in FVC, relative decline in FVC of 5% or more along with the increased extent of fibrosis on HRCT, relative decline in FVC of 5% or more along with worsening of respiratory symptoms or worsened respiratory symptoms and increased extent of fibrosis on HRCT. Recently, a definition of PF-ILD was proposed^[27]^ by adding the criteria of relative decline in FVC of 5% or more with a decline in DLco of 15% or more per 24 months to the INBUILD criteria.^[16]^ In this study, we defined PF-ILD according to the deterioration of the pulmonary function in the past 24 months.

In our study, 11 of 57 patients with non-IPF were diagnosed with ILD with a progressive phenotype (PF-ILD). Here, PF-ILDs included iNSIP, unclassifiable IIPs, pleuroparenchymal fibroelastosis, CTD-ILDs, and ANCA-related ILDs. Chronic hypersensitivity pneumonitis was not identified in this study; however, some cases of chronic hypersensitivity pneumonitis show a progressive phenotype. The diagnosis of chronic hypersensitivity pneumonitis is challenging,^[38]^ with varied diagnostic concordance across multidisciplinary teams.^[39]^ In this study, chronic hypersensitivity pneumonitis might have been diagnosed as IPF or unclassifiable IIPs in our multidisciplinary discussion.

First, in the present study, the pulmonary function at the first PFT was worse in the non-IPF, PF-ILD group than in the non-IPF, non-PF-ILD group. Low %FVC may be a risk factor for PF-ILD in the non-IPF group in addition to the UIP-like fibrotic pattern on HRCT. Thus, the pulmonologist should administer additional treatment before disease progression. Nintedanib has been shown to slow the FVC decline in non-IPF, PF-ILD patients;^[16]^ meaning, these patients can now be treated with antifibrotic agents. To avoid FVC decline, early treatment is essential.

Second, 6 of 11 patients (54.5%) in the non-IPF, PF-ILD group were observed without pharmacologic treatment, despite showing the deterioration of their pulmonary function. In these cases, there is a possibility that pharmacologic treatment was not administered due to concerns for adverse events of immunomodulation treatment. In addition, these patients may have only been observed because there was no effective treatment option so far. Nintedanib might be expected to be effective in these cases.

Third, the prognosis of patients with non-IPF, PF-ILD was as poor as that of IPF patients. Aging, low %DLco, and PF-ILD were risk factors for mortality in the non-IPF group. The prognosis of IPF patients in this study was better than that previously reported,^[5,6]^ which could be due to the difference in the inclusion criteria. The eligible patients in this study underwent PFT including DLco measurements twice or more for at least 24 months apart. Since patients with low FVC cannot undergo DLco assessments, ILD patients with rapid disease progression were not eligible for this study. Although the criteria were similar in the non-IPF group, IPF patients with rapid disease progression, that is, a poor prognosis, were not included in this study.

Our study had some limitations. First, several patients with rapid disease progression who could not undergo follow-up PFTs were not included in this study; therefore, their prognosis might have been better than the actual prognosis of the IPF and non-IPF, PF-ILD groups. Second, this was a single-center study with a small sample size. Recently, Nasser et al reported the clinical characteristics of patients with PF-ILD other than IPF in a real-world setting.^[40]^ They reported that 168 of 617 (27.2%) patients with non-IPF, ILD had progressive fibrosing phenotypes. This result was higher than our study; however, the definition of progressive phenotypes used in their study was based on INBUILD trial. Differences in the definition of progressive phenotypes might therefore have contributed to the difference in the results. Third, this was a retrospective study; thus, whether the management and assessment for disease behavior were proper or not was uncertain. A prospective study design with well-defined endpoints and a larger sample size is therefore advocated. Despite these limitations, we demonstrated the clinical characteristics of non-IPF, PF-ILD patients and showed that their prognosis was as poor as that of IPF patients.

In conclusion, the prognosis of patients with non-IPF, PF-ILD was as poor as that of IPF patients. Low %FVC may be a risk factor for PF-ILD in the non-IPF group. Non-IPF, PF-ILD patients require more intensive treatment before disease progression, and nintedanib might be an option.

## Acknowledgments

We would like to thank Editage (www.editage.jp) for English language editing.

## Author contributions

**Conceptualization:** Hiroshi Yamamoto, Masayuki Hanaoka.

**Data curation:** Masamichi Komatsu, Hiroshi Yamamoto, Yoshiaki Kitaguchi, Satoshi Kawakami, Mina Matsushita, Takeshi Uehara, Yosuke Wada, Takumi Kinjo, Takashi Ichiyama, Kazuhisa Urushihata, Atsuhito Ushiki, Masanori Yasuo.

**Formal analysis:** Masamichi Komatsu.

**Funding acquisition:** Masamichi Komatsu.

**Investigation:** Masamichi Komatsu.

**Methodology:** Masamichi Komatsu.

**Project administration:** Hiroshi Yamamoto.

**Supervision:** Hiroshi Yamamoto, Masayuki Hanaoka.

**Validation:** Masamichi Komatsu.

**Visualization:** Masamichi Komatsu.

**Writing – original draft:** Masamichi Komatsu.

**Writing – review & editing:** Hiroshi Yamamoto, Masayuki Hanaoka.
